# Antibiotic therapy in ventilator-associated tracheobronchitis: a literature review

**DOI:** 10.5935/0103-507X.20180014

**Published:** 2018

**Authors:** Abel Eduardo Alves, José Manuel Pereira

**Affiliations:** 1 Intensive Medicine Service, Hospital do Divino Espírito Santo - Ponta Delgada, Portugal.; 2 Intensive and Emergency Medicine Service, Centro Hospitalar de São João - Porto, Portugal.

**Keywords:** Bronchitis/diagnosis, Bronchitis/drug therapy, Bronchitis/epidemiology, Bronchitis/microbiology, Pneumonia, ventilator-associated/drug therapy, Tracheitis/diagnosis, Tracheitis/drug therapy, Tracheitis/epidemiology, Tracheitis/microbiology

## Abstract

The concept of ventilator-associated tracheobronchitis is controversial; its definition is not unanimously accepted and often overlaps with ventilator-associated pneumonia. Ventilator-associated tracheobronchitis has an incidence similar to that of ventilator-associated pneumonia, with a high prevalence of isolated multiresistant agents, resulting in an increase in the time of mechanical ventilation and hospitalization but without an impact on mortality. The performance of quantitative cultures may allow better diagnostic definition of tracheobronchitis associated with mechanical ventilation, possibly avoiding the overdiagnosis of this condition. One of the major difficulties in differentiating between ventilator-associated tracheobronchitis and ventilator-associated pneumonia is the exclusion of a pulmonary infiltrate by chest radiography; thoracic computed tomography, thoracic ultrasonography, or invasive specimen collection may also be required. The institution of systemic antibiotic therapy does not improve the clinical impact of ventilator-associated tracheobronchitis, particularly in reducing time of mechanical ventilation, hospitalization or mortality, despite the possible reduced progression to ventilator-associated pneumonia. However, there are doubts regarding the methodology used. Thus, considering the high prevalence of tracheobronchitis associated with mechanical ventilation, routine treatment of this condition would result in high antibiotic usage without clear benefits. However, we suggest the institution of antibiotic therapy in patients with tracheobronchitis associated with mechanical ventilation and septic shock and/or worsening of oxygenation, and other auxiliary diagnostic tests should be simultaneously performed to exclude ventilator-associated pneumonia. This review provides a better understanding of the differentiation between tracheobronchitis associated with mechanical ventilation and pneumonia associated with mechanical ventilation, which can significantly decrease the use of antibiotics in critically ventilated patients.

## INTRODUCTION

The definition of ventilator-associated tracheobronchitis (VAT) is not consensual. In patients ventilated for more than 48 hours, fever with no other cause and/or leukocyte count > 12,000/µL or < 4.000/µL associated with increased volume and/or purulence of respiratory secretions and endotracheal aspirate (ETA) with bacterial growth in the absence of a new pulmonary infiltrate or progression of previous infiltrate define this condition.^(1-3)^ Some authors consider the presence of purulent secretions (≥ 25 neutrophils and ≤ 10 squamous cells per small magnification field) mandatory,^(4)^ and the need for cultural examinations is not consensual.^(5)^ VAT is often considered an alternative diagnosis of ventilator-associated pneumonia (VAP).^(1)^

In this article, we review the published work on the use of antibiotic therapy in VAT. A search on the PubMed database was performed with the terms "ventilator", "tracheobronchitis", and "antibiotic" without time limitations. Manual search of relevant references cited in the selected papers was also performed. Only articles in English were considered.

### Epidemiology

Ventilator-associated tracheobronchitis has an incidence similar to that of VAP.^(2,4,6-8)^ In a recent meta-analysis^(9)^ with 3,362 patients, the incidence of VAT was 11.5%, ranging between 1.4%^(10)^ and 16.7%.^(7)^ According to the literature, it is possible to progress from VAT to VAP,^(4,6,8,10,11)^ and this phenomenon is reported in 12.2%^(4)^ to 34% of patients.^(11)^ A significant overlap between the two entities was not excluded at the time of diagnosis of VAT, a situation confirmed by the authors when they approached the limitations of the studies, namely, the difficulty in differentiating VAT and VAP by the analysis of chest radiography^(4,8,11)^ and the non-use of thoracic computed tomography.^(4)^

The prevalence of multiresistant agents implicated in VAT is high,^(4,10)^ leading to the early use of broad spectrum antibiotic therapy.^(4,12)^ The most frequently isolated agents are *Gram-*negative bacteria, mainly including *Pseudomonas aeruginosa, Klebsiella pneumoniae* and *Escherichia coli*. Equally relevant is the prevalence of *Staphylococcus aureus* despite significant variation between different intensive care units (ICU).^(4)^

Ventilator-associated tracheobronchitis increases the duration of mechanical ventilation and the length of stay in the ICU and hospital^(2,4,6,10,11,13-15)^ possibly because it hampers ventilatory weaning and extubation^(12)^ rather than the subsequent development of pneumonia.^(2)^ Given that heavily colonized patients with no infection in clinical examination did not present significant differences in these variables,^(6)^ VAT should be considered an independent clinical entity.^(16)^ According to Nseir,^(2)^ VAT significantly increases mortality in medical patients possibly due to subsequent evolution to VAP, but this finding has not been confirmed by other authors.^(4,9)^

### Pathophysiology

Tracheal intubation facilitates the entry of bacteria into the lower respiratory tract and subsequent colonization. Some authors^(12)^ advocate that a colonization process will occur from the established equilibrium between the bacterial virulence factors and the host defense mechanisms, which may evolve in some cases to VAT with purulent bronchiolitis associated with high counting of colonies or to VAP with alveolar pulmonary lesion. In addition, *postmortem* studies suggest the existence of a continuum between the tracheobronchial and pneumonic processes.^(17)^

### Diagnosis

The ETA collection for semiquantitative or quantitative culture examination is suggested by some authors^(2,3,6,12)^ as a strategy that allows a better diagnostic definition of the VAT. In this context, several studies address the importance of semiquantitative and quantitative cultures, namely, regarding the differentiation between heavily colonized patients and those with tracheobronchitis or pneumonia. No consensus exists regarding the reference values that should be used.^(2,6,18)^ However, only patients with bacterial growth ≥ 10^5^CFU/mL in the ETA have been included in the last published studies,^(4,6,8,10,11)^ and a good correlation exists between this threshold and semiquantitative cultures with moderate growth.^(6)^ However, there are several centers where quantitative or semiquantitative cultures are not routinely used as an integral component of the diagnosis of VAT,^(16)^ a situation that may lead to an overdiagnosis of this entity and that limits the extrapolation of results from clinical trials, most of which involve patient selection based on quantitative respiratory samples.

One of the major difficulties in the diagnosis of VAT is the exclusion of a new pulmonary infiltrate and consequently the differentiation of VAP. Previous studies have demonstrated that only 68% of VAP can be diagnosed by chest X-ray analysis,^(19)^ and thoracic computed tomography can significantly increase diagnostic sensitivity by approximately 44%.^(20)^ However, in a recently published multicenter survey,^(16)^ 49.2% of the participating centers reported never considering this possibility. Considering the frequent overlap of microbiological criteria in samples collected by ETA,^(3)^ the collection of respiratory samples by bronchoalveolar lavage (BAL) or protected bronchial brush (PBB) may also be important in the differentiation between VAT and VAP, namely, by not meeting criteria for the latter entity (bacterial growth ≥ 10^3^CFU/mL in PBB or ≥ 10^4^CFU/mL in BAL^(21)^). The non-use of antibiotics in patients with negative quantitative cultures collected by these methods appears to be safe.^(21-24)^

When performed by experienced professionals, thoracic ultrasound (TUS) also exhibits high sensitivity and specificity in the diagnosis of community pneumonia in non-ventilated patients^(25,26)^ and appears to increase diagnostic sensitivity in patients with clinical symptoms consistent with pneumonia and radiography of the chest not suggestive of this diagnosis.^(26)^ In a prospective study including 99 patients with clinic symptoms and chest X-ray suggestive of VAP, Mongodi et al.^(27)^ evaluated the utility of TUS in its early diagnosis. They concluded that the presence of subpleural consolidations and the presence of dynamic air bronchogram are ultrasound signals that exhibit high sensitivity and specificity for the diagnosis of VAP confirmed by bacterial growth ≥ 10^4^CFU /mL in BAL or by clinical criteria if BAL is negative under antibiotic therapy. In this context, TUS can help differentiate pneumonic from non-pneumonic infiltrates. However, additional studies are needed to confirm these results and clarify the usefulness of TUS in PAV/VAT differentiation, especially in patients with a chest X-ray not suggestive of VAP.

### To treat or not to treat with antibiotics

Table 1 summarizes the studies that addressed the impact of antibiotic therapy on the clinical course of VAT.

**Table 1:** Clinical impact of antibiotic therapy in the clinical course of ventilator-associated tracheobronchitis

	Study design	Quantitative cultures required for diagnosis	Results
Nseir et al.^(2)^	Observational prospective, single center	No	No statistically significant differences in the time of MV, length of stay in the ICU, or mortality (lower mortality in the medical subgroup of patients)
Martin-Loeches et al.^(4)^	Observational prospective, multicenter	Yes	No statistically significant differences in the time of MV, length of ICU stay, or mortality. Reduced progression to VAP
Nseir et al.^(8)^	Observational prospective, multicenter	Yes	Reduced progression to VAP. No mention of antibiotic therapy impact on other variables
Nseir et al.^(11)^	Randomized prospective, multicenter	Yes	No statistically significant differences in the time of MV or length of ICU stay. Significant differences were found for a greater number of days free of MV and reduced mortality in the ICU due to all causes. Reduced progression to VAP
Karvouniaris et al.^(13)^	Observational prospective, single center	Yes	No statistically significant difference in mortality. No mention of antibiotic therapy impact on other variables
Nseir et al.^(14)^	Observational prospective, single center	Yes	No statistically significant difference in the time of MV, length of ICU stay, or mortality.

MV - mechanical ventilation; ICU - intensive care unit; VAP - ventilator-associated pneumonia.

The institution of antibiotic therapy in VAT patients is not consensual, mainly in relation to its potential advantages.^(1,16)^ The assumptions in favor of this practice are based on a possible reduction in the time of mechanical ventilation and hospitalization and more recently in the reduction of the progression to VAP.^(4,8)^ It is unclear whether these occasional benefits are derived exclusively from VAT therapy, as it is possible that patients with early stage VAP are being treated for VAT.^(3)^

There is no evidence in the literature that the institution of antibiotic therapy in VAT patients results in a statistically significant reduction in the time of mechanical ventilation and hospitalization despite the statistically significant reduction of the bacterial inoculum.^(11)^ The possibility of antibiotic therapy reducing VAP progression is reported by some authors.^(4,8,11)^ It is limited not only by the low accuracy of the chest X-ray in the differentiation between the two entities but also by the non-use of other auxiliary diagnostic tests. In addition, the reduction in progression to documented PAV did not translate into reduced time for mechanical ventilation, hospitalization or mortality.

Some studies^(2,11)^ report a reduction in mortality with the institution of antibiotic therapy, although only in specific subgroups of patients^(2)^ or considering all-cause mortality.^(11)^ These findings, which were not reproduced in other studies,^(4,13,14)^ are controversial given that we do not find references in the literature suggesting that VAT conditions increase mortality regardless of the use of antibiotic therapy.

Considering the lack of clear benefits described in the literature, the associated costs, the risk of toxicity and the potential emergence of multiresistant microorganisms, we understand that antibiotic therapy should not be routinely used in patients with VAT despite its high prevalence. This view is shared by recently published clinical guidance standards.^(28)^ However, antibiotic therapy should be considered in VAT patients who present with septic shock and/or worsening of oxygenation and efforts should be made to exclude other differential diagnoses, such as VAP,^(28)^ a situation that may also permit a reduced duration of antibiotic therapy.

Despite increasing the time of ventilation and hospitalization, treatment with systemic antibiotics does not seem to alter the clinical course of VAT, possibly in relation to the low antibiotic concentration reached in the proximal upper airway.^(29)^ Therefore, the use of inhaled antibiotic therapy in such cases has been suggested. In fact, inhaled antibiotics, providing high concentrations in the respiratory tract with minimal systemic effects,^(30)^ exhibited promising results in the reduction of bacterial inoculum^(31-33)^ with a possible reduction of the risk of developing bacterial resistance. These data should be confirmed in subsequent studies.^(30,34)^

**Figure 1:**
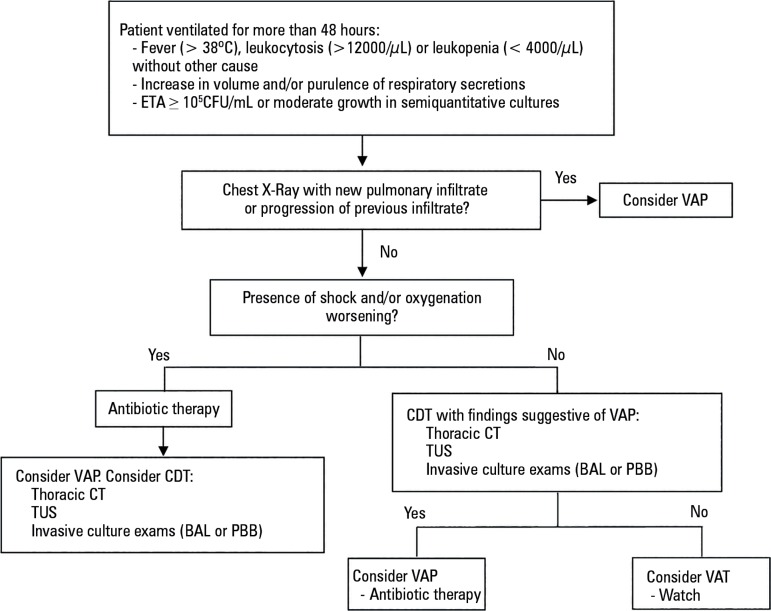
Algorithm to approach a patient with suspected ventilator-associated tracheobronchitis. ETA - endotracheal aspirate; CFU - colony forming units; VAP - ventilator-associated pneumonia; CDT - complementary diagnostic tests; CT - computed tomography; TUS - thoracic ultrasonography; BAL - bronchoalveolar lavage; PBB - protected bronchial brush; VAT - ventilator-associated tracheobronchitis.

In figure 1, an algorithm to approach a patient with suspected VAT is proposed.

## CONCLUSION

Respiratory infections account for half of the antibiotic prescriptions in intensive care. Although some authors recommend the use of antibiotic therapy in ventilator-associated tracheobronchitis to reduce eventual progression to ventilator-associated pneumonia, the methodology used does not exclude a significant diagnostic overlap. Furthermore, its use does not lead to a statistically significant reduction in mechanical ventilation time, length of hospital stay, or mortality.

Considering the high prevalence of ventilator-associated tracheobronchitis and the consequent significant antibiotic prescription associated with its routine treatment, we suggest that antibiotic therapy should be considered only for patients with ventilator-associated tracheobronchitis with septic shock and/or oxygenation deficiency. In these cases, given that the probability of being ventilator-associated pneumonia is high, we suggest the use of other diagnostic auxiliary exams for its exclusion.

Considering the limitations of the published studies, additional studies are needed with differentiating criteria between the two entities that exclude a significant diagnostic overlap to reliably evaluate the possible benefit of systemic antibiotic therapy in ventilator-associated tracheobronchitis.

## References

[1] American Thoracic Society Infectious Diseases Society of America (2005). Guidelines for the management of adults with hospital-acquired,
ventilator-associated, and healthcare-associated pneumonia. Am J Respir Crit Care Med.

[2] Nseir S, Di Pompeo C, Pronnier P, Beague S, Onimus T, Saulnier F (2002). Nosocomial tracheobronchitis in mechanically ventilated patients:
incidence, aetiology and outcome. Eur Respir J.

[3] Craven DE, Chroneou A, Zias N, Hjalmarson KI (2009). Ventilator-associated tracheobronchitis: the impact of targeted
antibiotic therapy on patient outcomes. Chest.

[4] Martin-Loeches I, Povoa P, Rodríguez A, Curcio D, Suarez D, Mira JP, Cordero ML, Lepecq R, Girault C, Candeias C, Seguin P, Paulino C, Messika J, Castro AG, Valles J, Coelho L, Rabello L, Lisboa T, Collins D, Torres A, Salluh J, Nseir S, TAVeM study (2015). Incidence and prognosis of ventilator-associated
tracheobronchitis (TAVeM): a multicentre, prospective, observational
study. Lancet Respir Med.

[5] Torres A, Ewig S, Lode H, Carlet J, European HAP working group (2009). Defining, treating and preventing hospital acquired pneumonia:
European perspective. Intensive Care Med.

[6] Craven DE, Lei Y, Ruthazer R, Sarwar A, Hudcova J (2013). Incidence and outcomes of ventilator-associated tracheobronchitis
and pneumonia. Am J Med.

[7] Shahin J, Bielinski M, Guichon C, Flemming C, Kristof AS (2013). Suspected ventilator-associated respiratory infection in severely
ill patients: a prospective observational study. Crit Care.

[8] Nseir S, Martin-Loeches I, Makris D, Jaillette E, Karvouniaris M, Valles J (2014). Impact of appropriate antimicrobial treatment on transition from
ventilator-associated tracheobronchitis to ventilator-associated
pneumonia. Crit Care.

[9] Agrafiotis M, Siempos II, Falagas ME (2010). Frequency, prevention, outcome and treatment of
ventilator-associated tracheobronchitis: systematic review and
meta-analysis. Respir Med.

[10] Dallas J, Skrupky L, Abebe N, Boyle 3rd WA, Kollef MH (2011). Ventilator-associated tracheobronchitis in a mixed surgical and
medical ICU population. Chest.

[11] Nseir S, Favory R, Jozefowicz E, Decamps F, Dewavrin F, Brunin G, Di Pompeo C, Mathieu D, Durocher A, VAT Study Group (2008). Antimicrobial treatment for ventilator-associated
tracheobronchitis: a randomized, controlled, multicenter
study. Crit Care.

[12] Craven DE, Hjalmarson KI (2010). Ventilator-associated tracheobronchitis and pneumonia: thinking
outside the box. Clin Infect Dis.

[13] Karvouniaris M, Makris D, Manoulakas E, Zygoulis P, Mantzarlis K, Triantaris A (2013). Ventilator-associated tracheobronchitis increases the length of
intensive care unit stay. Infect Control Hosp Epidemiol.

[14] Nseir S, Di Pompeo C, Soubrier S, Lenci H, Delour P, Onimus T (2005). Effect of ventilator-associated tracheobronchitis on outcome in
patients without chronic respiratory failure: a case-control
study. Crit Care.

[15] Nseir S, Ader F, Marquette CH (2009). Nosocomial tracheobronchitis. Curr Opin Infect Dis.

[16] Rodríguez A, Póvoa P, Nseir S, Salluh J, Curcio D, Martín-Loeches I, TAVeM group investigators (2014). Incidence and diagnosis of ventilator-associated
tracheobronchitis in the intensive care unit: an international online
survey. Crit Care.

[17] Nseir S, Marquette CH (2003). Diagnosis of hospital-acquired pneumonia: postmortem
studies. Infect Dis Clin North Am.

[18] Dallas J, Kollef M (2009). VAT vs VAP: are we heading toward clarity or
confusion?. Chest.

[19] Wunderink RG, Woldenberg LS, Zeiss J, Day CM, Ciemins J, Lacher DA (1992). The radiologic diagnosis of autopsy-proven ventilator-associated
pneumonia. Chest.

[20] Syrjälä H, Broas M, Suramo I, Ojala A, Lähde S (1998). High-resolution computed tomography for the diagnosis of
community-acquired pneumonia. Clin Infect Dis.

[21] Fagon JY, Chastre J, Wolff M, Gervais C, Parer-Aubas S, Stéphan F (2000). Invasive and noninvasive strategies for management of suspected
ventilator-associated pneumonia. A randomized trial. Ann Intern Med.

[22] Croce MA, Fabian TC, Shaw B, Stewart RM, Pritchard FE, Minard G (1994). Analysis of charges associated with diagnosis of nosocomial
pneumonia: can routine bronchoscopy be justified?. J Trauma.

[23] Bonten MJ, Bergmans DC, Stobberingh EE, van der Geest S, De Leeuw PW, van Tiel FH (1997). Implementation of bronchoscopic techniques in the diagnosis of
ventilator-associated pneumonia to reduce antibiotic use. Am J Respir Crit Care Med.

[24] Heyland DK, Cook DJ, Marshall J, Heule M, Guslits B, Lang J (1999). The clinical utility of invasive diagnostic techniques in the
setting of ventilator-associated pneumonia. Canadian Critical Care Trials
Group. Chest.

[25] Reissig A, Copetti R, Mathis G, Mempel C, Schuler A, Zechner P (2012). Lung ultrasound in the diagnosis and follow-up of
community-acquired pneumonia: a prospective, multicenter, diagnostic
accuracy study. Chest.

[26] Parlamento S, Copetti R, Di Bartolomeo S (2009). Evaluation of lung ultrasound for the diagnosis of pneumonia in
the ED. Am J Emerg Med.

[27] Mongodi S, Via G, Girard M, Rouquette I, Misset B, Braschi A (2016). Lung ultrasound for early diagnosis of ventilator-associated
pneumonia. Chest.

[28] Kalil AC, Metersky ML, Klompas M, Muscedere J, Sweeney DA, Palmer LB (2016). Management of Adults with Hospital-acquired and
Ventilator-associated Pneumonia: 2016 Clinical Practice Guidelines by the
Infectious Diseases Society of America and the American Thoracic
Society. Clin Infect Dis.

[29] Mendelman PM, Smith AL, Levy J, Weber A, Ramsey B, Davis RL (1985). Aminoglycoside penetration, inactivation, and efficacy in cystic
fibrosis sputum. Am Rev Respir Dis.

[30] Russell CJ, Shiroishi MS, Siantz E, Wu BW, Patino CM (2016). The use of inhaled antibiotic therapy in the treatment of
ventilator-associated pneumonia and tracheobronchitis: a systematic
review. BMC Pulm Med.

[31] Palmer LB, Smaldone GC, Chen JJ, Baram D, Duan T, Monteforte M (2008). Aerosolized antibiotics and ventilator-associated
tracheobronchitis in the intensive care unit. Crit Care Med.

[32] Athanassa ZE, Myrianthefs PM, Boutzouka EG, Tsakris A, Baltopoulos GJ (2011). Monotherapy with inhaled colistin for the treatment of patients
with ventilator-associated tracheobronchitis due to
polymyxin-only-susceptible Gram-negative bacteria. J Hosp Infect.

[33] Palmer LB, Smaldone GC (2014). Reduction of bacterial resistance with inhaled antibiotics in the
intensive care unit. Am J Respir Crit Care Med.

[34] Palmer LB (2015). Ventilator-associated infection: the role for inhaled
antibiotics. Curr Opin Pulm Med.

